# Travelling-wave analysis of a model of tumour invasion with degenerate, cross-dependent diffusion

**DOI:** 10.1098/rspa.2021.0593

**Published:** 2021-12

**Authors:** Chloé Colson, Faustino Sánchez-Garduño, Helen M. Byrne, Philip K. Maini, Tommaso Lorenzi

**Affiliations:** ^1^ Wolfson Centre for Mathematical Biology, Mathematical Institute, University of Oxford, Radcliffe Observatory Quarter, Oxford OX2 6GG, UK; ^2^ Departamento de Matemáticas, Facultad de Ciencias, UNAM, Ciudad Universitaria, Circuito Exterior, Cd. de México, C.P. 04510, Mexico; ^3^ Department of Mathematical Sciences ‘G. L. Lagrange’, Politecnico di Torino, 10129 Torino, Italy

**Keywords:** travelling-wave solutions, degenerate and cross-dependent diffusion, tumour invasion

## Abstract

In this paper, we carry out a travelling-wave analysis of a model of tumour invasion with degenerate, cross-dependent diffusion. We consider two types of invasive fronts of tumour tissue into extracellular matrix (ECM), which represents healthy tissue. These types differ according to whether the density of ECM far ahead of the wave front is maximal or not. In the former case, we use a shooting argument to prove that there exists a unique travelling-wave solution for any positive propagation speed. In the latter case, we further develop this argument to prove that there exists a unique travelling-wave solution for any propagation speed greater than or equal to a strictly positive minimal wave speed. Using a combination of analytical and numerical results, we conjecture that the minimal wave speed depends monotonically on the degradation rate of ECM by tumour cells and the ECM density far ahead of the front.

## 1. Introduction

Tissue invasion is a hallmark of malignant tumours [1] and a classical mathematical approach to study this process involves reaction–diffusion (R–D) partial differential equations (PDEs) [2–4]. A key feature of many such models of tumour invasion is the inclusion of degenerate, cross-dependent diffusion. The aim of this paper is to study this common characteristic by proposing a minimal model which captures the main components of the tumour invasion process and is analytically tractable. We seek two types of constant profile, constant speed travelling-wave solutions (TWS) for our model. Both types represent invasive fronts of tumour tissue into extracellular matrix (ECM), which represents healthy tissue, but they differ according to whether the density of ECM far ahead of the wave front is maximal or not. For the former, we prove the existence and uniqueness of TWS for all positive propagation speeds using the shooting argument developed by Gallay & Mascia [5]. For the latter, we expand this shooting argument to prove the existence and uniqueness of TWS for propagation speeds greater than or equal to a strictly positive minimal value. Finally, we characterize this minimal wave speed using a conjecture motivated by a combination of analytical results and numerical simulations.

### (a) Reaction–diffusion PDE models of tumour invasion

To invade the surrounding healthy tissue, a tumour must overcome the defences developed by the body to maintain homeostatic control. An important barrier to tumour invasion is the ECM, which is a strong scaffold of proteins that holds tissue cells in place and initiates signalling pathways for cellular processes such as migration, differentiation and proliferation [6,7]. The healthy cells encased by the ECM form another barrier to invasion by creating a competitive environment for the tumour cells. However, tumour cells have developed mechanisms to overcome both of these barriers. First, they can remodel or degrade the ECM by producing specific matrix-degrading enzymes, which act in close proximity to the cells producing them [8,9]. Second, by favouring glycolytic metabolism even in aerobic conditions (i.e. the ‘Warburg effect’), tumour cells may acidify the tissue microenvironment, resulting in healthy cell death [10,11]. Matrix remodelling is a very localized process, in contrast to the diffusion of lactic acid, which occurs on a longer spatial range.

The pioneering model by Gatenby & Gawlinksi [2] describes the spatio-temporal dynamics of acid-mediated tumour invasion by considering the interactions of healthy tissue, tumour tissue and the lactic acid produced by the tumour cells. Denoting the dimensionless tumour and healthy tissue densities and acid concentration by N(x,t), M(x,t) and L(x,t), respectively, for (x,t)∈R×(0,+∞), their model takes the form

1.1 $$\begin{array}{rl} & \frac{\partial N}{\partial t}=\beta N(1-N)+\frac{\partial }{\partial x}\left[D_{N}(1-M)\frac{\partial N}{\partial x}\right],\\ & \frac{\partial M}{\partial t}=M(1-M-\alpha L)\\ \text{and}\;\; & \frac{\partial L}{\partial t}=\gamma (N-L)+\frac{\partial^{2}L}{\partial x^{2}}. \end{array}\}$$

Here, it is assumed that healthy cells do not move, while tumour cells can invade in a density-dependent manner. Depending on the value of α, the model describes the total or partial destruction of normal tissue following tumour invasion. We refer the reader to the original paper for full details of the model. A numerical study of the TWS of system (1.1), with $0<D_{N}\ll 1$, showed the existence of an *interstitial gap*, i.e. a region devoid of cells, formed locally ahead of the invading tumour front, for large values of α [2]. Experimental evidence has confirmed that such an interstitial gap can exist and, in this way, the model has led to novel and accurate predictions regarding tumour invasion. This is one of the reasons why this model and its variations have been widely investigated [3,12–16].

### (b) Nonlinear, degenerate diffusion: from scalar to multi-dimensional analysis

A key common component of the Gatenby–Gawlinksi model and its variations is the degenerate, cross-diffusion term in the equation for the tumour cell density. For scalar R–D equations with nonlinear, degenerate diffusion, TWS have been extensively studied; see for instance [17–25]. In general, if the dimensionless equation has a reaction term, f, of Fisher–Kolmogoroff–Petrovsky–Piscounoff (Fisher–KPP) type, i.e. f∈C[0,1] with f(0)=f(1)=0 and f(n)>0 ∀ n∈(0,1), then TWS exist and are unique if and only if the wave speed is greater than or equal to a minimal speed, $c^{*}>0$, defined as the threshold speed below which no TWS exist. Further, if $c=c^{*}$, then the TWS is of sharp type (that is, there is a discontinuity in the spatial derivative at the front) and, for each $c>c^{*}$, there exists a TWS of front type (that is, smooth). It is non-trivial to extend such an existence result to R–D systems with multiple equations because of the added complexity of studying trajectories in a phase space rather than a phase plane. Kawasaki *et al.* [26] do so for an R–D system with cross-dependent diffusion developed to describe spatio-temporal pattern formation in colonies of bacteria. More specifically, numerical and analytical investigations [25,27] have shown the existence of TWS for wave speeds above or equal to a critical value, $c^{*}>0$. Until recently, most comprehensive results on the existence of TWS for spatially resolved models of tumour invasion focused on models in which invasion is driven by haptotaxis or chemotaxis [28–31]. In particular, the existence of TWS for the Gatenby–Gawlinski model has been largely supported by a combination of numerical and analytical results [12–15,32,33]. This also holds for a simplified model of invasion by Browning *et al.* [34], as seen in [35]. However, key existence results were recently proved by Gallay & Mascia [5] for a reduced version of the Gatenby–Gawlinski model.

### (c) The mathematical model

We now present a minimal model of tumour invasion. There is increasing evidence that phenotypically heterogeneous tumours can contain sub-populations of cells with different traits, e.g. matrix-degrading cells and acid-producing cells [16]. Therefore, we make the simplifying assumption that the healthy tissue compartment solely comprises ECM, disregarding healthy cells, and we focus on the interactions of ECM-degrading tumour cells and ECM. Using a standard law for conservation of mass and denoting the tumour cell and ECM densities by N(x,t) and M(x,t), respectively, for (x,t)∈R×(0,+∞), we propose the following system of PDEs:

1.2 $$\begin{array}{rl} & \frac{\partial N}{\partial t}=\underset{\text{tumour cell movement}}{\underbrace{\frac{\partial }{\partial x}\left[D_{N}\left(1-\frac{M}{M_{\text{Max}}}\right)\frac{\partial N}{\partial x}\right]}}+\underset{\text{tumour growth}}{\underbrace{\rho \left(1-\frac{N}{K}\right)N}}\\ \text{and}\;\; & \frac{\partial M}{\partial t}=\underset{\text{ECM degradation}}{\underbrace{-kMN}}. \end{array}\}$$

We assume that the tumour grows logistically, with maximum growth rate ρ and carrying capacity K. Further, the ECM acts as a physical barrier that inhibits tumour cell movement, but not proliferation. Thus, following Gatenby & Gawlinski [2] and others [3,16,36], we define the diffusivity of tumour cells as a monotonically decreasing function of the ECM density to model the obstruction of movement by the ECM. The diffusivity of tumour cells in the absence of ECM is denoted by $D_{N}$ and the ECM density that inhibits all tumour cell movement is denoted by $M_{\text{Max}}$. Finally, we assume that the ECM does not grow and is degraded at a rate that is proportional to the local tumour cell density, with a per cell degradation rate of k. We use a mass-action term to reflect the localized nature of matrix degradation.

To reduce the number of free parameters in the system and facilitate the analysis that follows, we non-dimensionalize equations (1.2) and, retaining the same dimensional state variables for notational convenience, we obtain the following system:

1.3 $$\begin{array}{rl} & \frac{\partial N}{\partial t}=\frac{\partial }{\partial x}\left[(1-M)\frac{\partial N}{\partial x}\right]+(1-N)N\\ \text{and}\;\; & \frac{\partial M}{\partial t}=-\kappa MN, \end{array}\}$$

where κ=(K/ρ)k.

Here, we note that system (1.3) is similar to a reduced version of the model (1.1) from Moschetta & Simeoni [15], studied by Gallay & Mascia [5], and a reduced model of melanoma invasion from Browning *et al.* [34], studied by El Hachem *et al.* [35]. In particular, while the models differ according to the reaction terms included for the healthy and tumour cell densities, each model retains the same degeneracy in the cross-diffusion term, which is the key focus of this paper.

Gallay & Mascia [5] rigorously proved the existence of a weak form of TWS for any positive wave speed, c>0, for the model in [15]. These TWS represent the invasion of tumour tissue into healthy tissue, where the density of healthy tissue ahead of the wave front is at carrying capacity. El Hachem *et al.* [35] performed a numerical study which suggests that such TWS also exist for the model in [34] for any positive wave speed. In addition, their numerical results indicated the existence of another type of TWS for the model in [34] for wave speeds above a strictly positive minimal value. These TWS differ from the former in that the density of healthy tissue ahead of the wave front is below carrying capacity. Finally, El Hachem *et al.* [35] described the dependence of the minimal wave speed of this second type of TWS on the rescaled degradation rate of healthy tissue, which we denote by κ: it remains constant provided κ is below some threshold value, $\kappa^{*}$, which is yet to be determined, and then increases with κ for $\kappa \geq \kappa^{*}$.

The key contribution of the present paper is to rigorously prove the existence of both aforementioned types of TWS for system (1.3), which we achieve by applying and expanding the shooting argument developed by Gallay & Mascia [5]. Similarly to [5,35], we will find that the first type of TWS exists for all c>0, whereas there is a strictly positive minimal wave speed for the second type of TWS. We will see that this minimal wave speed for TWS of system (1.3) can be qualitatively characterized in the same way as that for equivalent TWS of the model in [34]. However, given κ, the value of this minimal wave speed for TWS of system (1.3) and of the system in [34] is not the same. A final contribution of our work compared with that in [35] is that we propose an expression for $\kappa^{*}$ for system (1.3), not reported in [35] for the model in [34].

### (d) Structure of the paper

We will seek constant profile, constant speed TWS for (1.3), which are heteroclinic trajectories of a three-dimensional dynamical system connecting two of its steady states. These correspond to spatially homogeneous, steady-state solutions of (1.3), which are given by

1.4 $$(N_{0}^{*},M_{0}^{*})=(0,0),\;(N_{1}^{*},M_{1}^{*})=(1,0),\;(N_{2}^{*},M_{2}^{*})=(0,1),\;(N_{3}^{*},M_{3}^{*})=(0,\bar{M}),\;\bar{M}\in [0,1).$$

Here, $\left(N_{0}^{*},M_{0}^{*}\right)$ is the trivial state, $\left(N_{1}^{*},M_{1}^{*}\right)$ is a state in which the tumour has successfully invaded and degraded all ECM and $\left(N_{2}^{*},M_{2}^{*}\right)$ and $\left(N_{3}^{*},M_{3}^{*}\right)$ are, respectively, a tumour-free state at maximum ECM density and a continuum of tumour-free states. We distinguish $\left(N_{2}^{*},M_{2}^{*}\right)$ from $\left(N_{3}^{*},M_{3}^{*}\right)$ because of the degeneracy at M=1 in system (1.3). Since we are interested in studying the existence of TWS that describe the invasion of a tumour into healthy tissue, we will look for two types of heteroclinic trajectories: those connecting $\left(N_{1}^{*},M_{1}^{*}\right)$ to $\left(N_{2}^{*},M_{2}^{*}\right)$ and those connecting $\left(N_{1}^{*},M_{1}^{*}\right)$ to $\left(N_{3}^{*},M_{3}^{*}\right)$. In §2, we define the TWS we seek, prove preliminary results and derive the ordinary differential equation (ODE) system they must satisfy. In §3, we use the shooting argument developed by Gallay & Mascia [5] to show that system (1.3) has a unique TWS connecting $\left(N_{1}^{*},M_{1}^{*}\right)$ to $\left(N_{2}^{*},M_{2}^{*}\right)$ for any positive wave speed. We then show that, for each $\bar{M}\in [0,1)$, system (1.3) has a unique TWS connecting $\left(N_{1}^{*},M_{1}^{*}\right)$ to $\left(N_{3}^{*},M_{3}^{*}\right)$ for any wave speed greater than or equal to a strictly positive minimum value. Motivated by our numerical simulations and partial analytical results, we make a conjecture about the dependence of the minimal wave speed on $\bar{M}\in [0,1)$ and κ>0, the rescaled degradation rate of the ECM. In §4, we present numerical simulations of system (1.3) which support and complement the preceding analytical results. We conclude the paper in §5, where we discuss our results alongside future research perspectives.

## 2. The travelling-wave problem

### (a) Preliminaries

We seek constant profile, constant speed TWS of system (1.3) by introducing the travelling-wave coordinate ξ=x−ct. We require the wave speed c>0 so that the tumour invades the ECM from left to right in the spatial domain. Substituting the ansatz N(x,t)=N(ξ) and M(x,t)=M(ξ) into system (1.3), we deduce that TWS must satisfy the following ODE system:

2.1a $$\frac{\text{d}}{\text{d}\xi }\left((1-M)\frac{\text{d}N}{\text{d}\xi }\right)+c\frac{\text{d}N}{\text{d}\xi }+(1-N)N=0$$

and

2.1b $$c\frac{\text{d}M}{\text{d}\xi }-\kappa MN=0.$$

The TWS we seek connect spatially homogeneous steady states of system (1.3) and, equivalently, steady states of system (2.1*a*,b). Thus, we require one of the following sets of asymptotic conditions to be satisfied:

2.2 $$\lim_{\xi \to -\infty }(N(\xi ),M(\xi ))=(1,0),\;\lim_{\xi \to +\infty }(N(\xi ),M(\xi ))=(0,1)$$

or

2.3 $$\lim_{\xi \to -\infty }(N(\xi ),M(\xi ))=(1,0),\;\lim_{\xi \to +\infty }(N(\xi ),M(\xi ))=(0,\bar{M})\;\text{with }\bar{M}\in [0,1).$$

In other words, far behind the wave, the tumour density is at carrying capacity and the ECM has been fully degraded, whereas, far ahead of the wave, the tumour density is zero and the ECM density is either at carrying capacity (i.e. M=1) or at any value M∈[0,1). As noted previously, the first equation in system (1.3) is a degenerate parabolic equation since the cross-diffusion coefficient D(M)=1−M is zero when M=1. The existence of global classical solutions of this PDE system and the corresponding ODE system (2.1*a*,b) is therefore unclear in cases where M=1 or, correspondingly, where M=1. We therefore define a weak TWS in a similar way to the definition of a propagation front in [5].

**Definition 2.1.**

The triple (N,M;c) is called a weak TWS for system (1.3) if

(i) (N,M)∈C(R,[0,1])×C(R,[0,1]) and $\left(1-M)(\text{d}N/\text{d}\xi )\in L^{2}(R\right)$;
(ii) (N,M) is a weak solution of (2.1*a*,b), i.e. for all $\left(\phi ,\psi )\in C^{1}(R)\times C^{1}(R\right)$ with compact support
  2.4 $$\int_{R}\left\{\left[cN+(1-M)\frac{\text{d}N}{\text{d}\xi }\right]\frac{\text{d}\phi }{\text{d}\xi }-(1-N)N\phi \right\}\;d\xi =0$$
  and
  2.5 $$\int_{R}M\left\{c\frac{\text{d}\psi }{\text{d}\xi }+\kappa N\psi \right\}\;d\xi =0;$$
(iii) one of the pairs of asymptotic conditions given by (2.2) and (2.3), respectively, are satisfied.

We refer to (N,M) as the travelling-wave profile and c as the propagation speed.

**Note.**

Hence, unless otherwise stated, we refer to weak TWS in the sense of definition 2.1 as TWS.

If (N,M;c) is a TWS for system (1.3), then we can show that $N(1-N)\in L^{1}(R)$ and c>0 using a proof identical to that of lemma 2.1 in [5] and, thus, we omit it. The following lemma, whose proof follows as in [5], states that if (N,M;c) is a TWS for system (1.3), then N and M are non-negative and bounded and, thus, the TWS is biologically realistic.

**Lemma 2.2.**

*If* (N,M;c) *is a weak TWS, in the sense of definition* 2.1, *that satisfies the asymptotic conditions* (2.3) *for* $\bar{M}\in (0,1)$, *then there exists a unique point* $\bar{\xi }\in R\cup \{+\infty \}$ *such that*

(i) $N,M\in C^{\infty }((-\infty ,\bar{\xi }))$ *and* 0<N(ξ)<1, $0<M(\xi )<\bar{M}$ *for* $\xi <\bar{\xi }$;
(ii) *if* $\bar{\xi }<+\infty$, *then* N(ξ)=0 *and* $M(\xi )=\bar{M}$ *for all* $\xi \geq \bar{\xi }.$

**Remark 2.3.**

The case of TWS that satisfy the asymptotic conditions (2.3) for $\bar{M}=0$ is not considered in lemma 2.2. By definition, such solutions satisfy $\lim_{\xi \to \pm \infty }M(\xi )=0$ for N≥0, which is only possible if M≡0 on R since M is increasing for N,M>0. In this case, system (2.1*a*,b) reduces to the Fisher–KPP equation, which has been extensively studied [37–39]. It is known that the Fisher–KPP equation admits classical TWS that satisfy the asymptotic conditions $\lim_{\xi \to -\infty }N(\xi )=1$, $\lim_{y\to +\infty }N(\xi )=0$ and $\lim_{\xi \to \pm \infty }(\text{d}N/\text{d}\xi )(\xi )=0$ for all c≥2. This result, therefore, holds for TWS of (2.1*a*,b) satisfying the asymptotic conditions (2.3) for $\bar{M}=0$.

A version of lemma 2.2 for TWS that satisfy the asymptotic conditions (2.2) follows similarly [5]. These results highlight that the solutions we seek are classical solutions of system (2.1*a*,b) on intervals of the form $\left(-\infty ,\bar{\xi }\right)$. Further, if $\bar{\xi }=+\infty$, then the TWS are here called *smooth*. In contrast, if $\bar{\xi }<+\infty$, then lemma 2.2 implies that we have a corner point at $\bar{\xi }$ and the TWS are here called *sharp*.

### (b) Desingularization of the ODE system

Definition 2.1 describes two types of TWS of system (2.1*a*,b), which differ in the asymptotic conditions they satisfy at infinity. One type of solution converges to (N,M)=(0,1) at infinity. Therefore, we need to elucidate the behaviour of solutions as they approach M=1, which is precisely when system (2.1*a*,b) is *singular*. A common approach to simplify the analysis is to remove this singularity by re-parametrizing the system. Given a solution (N,M) of system (2.1*a*,b) satisfying either (2.2) or (2.3), we introduce a new independent variable y=Φ(ξ) defined such that

2.6 $$\frac{\text{d}y}{\text{d}\xi }\equiv \Phi^{\prime }(\xi )=\frac{1}{1-M(\xi )}\;\forall \;\xi \in R.$$

Further introducing the following dependent variables:

2.7 $$n(y)=N(\Phi^{-1}(y)),\;m(y)=M(\Phi^{-1}(y)),\;y\in R,$$

we can apply the chain rule and use (2.6) to find that, for 0≤m≤1, the trajectories satisfy the following ODE system, for y∈R:

2.8a $$\frac{d^{2}n}{dy^{2}}+c\frac{\text{d}n}{\text{d}y}+(1-n)n(1-m)=0$$

and

2.8b $$\frac{\text{d}m}{\text{d}y}-\frac{\kappa }{c}m(1-m)n=0.$$

In line with the asymptotic conditions (2.2) and (2.3), we require one of the following to hold:

2.9 $$\lim_{y\to -\infty }(n(y),m(y))=(1,0),\;\lim_{y\to +\infty }(n(y),m(y))=(0,1)$$

or

2.10 $$\lim_{y\to -\infty }(n(y),m(y))=(1,0),\;\lim_{y\to +\infty }(n(y),m(y))=(0,\bar{m})\;\text{with }\bar{m}\in [0,1).$$

Importantly, system (2.8*a*,b) is topologically equivalent to system (2.1*a*,b) for $(N,M)\in {\left(0,1\right)}^{2}$. This follows from the fact that (2.7) defines a homeomorphism that maps the orbits of (2.1*a*,b) onto the orbits of (2.8*a*,b), while preserving their orientation—(2.6) implies that y is an increasing function of ξ for all 0≤M<1. We also observe that, in contrast to system (2.1*a*,b), system (2.8*a*,b) has an additional continuum of steady states of the form $\left(n,m)=(\bar{n},1\right)$, $\bar{n}\in (0,1]$. These are not spatially homogeneous steady states of the original PDE system (1.3), so we do not consider them as asymptotic conditions in the context of TWS.

We finally obtain a system of three first-order ODEs by introducing the additional variable p=dn/dy and, using primes to denote derivatives with respect to y, we have

2.11a $$\begin{array}{rl} & n^{\prime }=p, \end{array}$$

2.11b $$\begin{array}{rl} & p^{\prime }=-cp-(1-n)n(1-m) \end{array}$$

2.11c $$\begin{array}{rl} \text{and}\;\; & m^{\prime }=\frac{\kappa }{c}m(1-m)n. \end{array}$$

In the following section, we set up a framework, first proposed in [5] for a different system, to study two distinct types of solutions of (2.11*a*–*c*). First, those that remain in the region $D_{1}$, defined as

2.12 $$D_{1}:=\{(n,p,m)\in R^{3}|m\in (0,1),n\in (0,1),p\in (-\infty ,0)\},$$

and that satisfy $\lim_{y\to -\infty }(n(y),p(y),m(y))=(1,0,0)$, $\lim_{y\to +\infty }(n(y),p(y),m(y))=(0,0,1)$. Second, for $\bar{m}\in (0,1)$, those that remain in the region $D_{\bar{m}}$, defined similarly to (2.12) as

2.13 $$D_{\bar{m}}:=\{(n,p,m)\in R^{3}|m\in (0,\bar{m}),n\in (0,1),p\in (-\infty ,0)\},$$

and that satisfy $\lim_{y\to -\infty }(n(y),p(y),m(y))=(1,0,0)$, $\lim_{y\to +\infty }(n(y),p(y),m(y))=(0,0,\bar{m})$.

## 3. Travelling-wave analysis

In this section, we study the existence of TWS. To do so, we apply the shooting argument developed by Gallay & Mascia [5]. The crucial difference between Gallay & Mascia’s model and system (1.3) is that the latter has an additional continuum of steady states of the form $\left(0,\bar{M}\right)$, $\bar{M}\in (0,1)$. We find that the results of [5] for TWS connecting the equilibrium points (1,0,0) and (0,0,1) apply, with minor modifications, to the TWS of system (2.11*a*–*c*) that satisfy the same asymptotic conditions (2.2). Therefore, in what follows, we state the key results and present only those proofs which require a different approach. For TWS of system (2.11*a*–*c*) that satisfy the asymptotic conditions (2.3), we further develop the shooting argument to obtain new results.

### (a) Local analysis of the equilibrium point (1,0,0): defining the shooting parameter

The TWS of interest satisfy $\lim_{y\to -\infty }(n(y),p(y),m(y))=(1,0,0)$. We therefore study the behaviour of solutions of (2.11*a*–*c*) in a neighbourhood of the equilibrium point $P_{1}:=(1,0,0)$ by performing a linear stability analysis. The Jacobian matrix at $P_{1}$ reduces to

$$J{|}_{\left(1,0,0\right)}=\left[\begin{array}{ccc} 0 & 1 & 0\\ 1 & -c & 0\\ 0 & 0 & \frac{\kappa }{c} \end{array}\right],$$

and it has the following eigenvalues and eigenvectors:

3.1 $$\lambda_{1}=\frac{-c-\sqrt{c^{2}+4}}{2},\;\lambda_{2}=\frac{-c+\sqrt{c^{2}+4}}{2},\;\lambda_{3}=\frac{\kappa }{c}$$

and

3.2 $$v_{1}={\left(\frac{c-\sqrt{c^{2}+4}}{2},1,0\right)}^{⊤},\;v_{2}={\left(\frac{c+\sqrt{c^{2}+4}}{2},1,0\right)}^{⊤},\;v_{3}={\left(0,0,1\right)}^{⊤}.$$

Since $\lambda_{1}$ is negative and $\lambda_{2}$ and $\lambda_{3}$ are positive, $P_{1}$ is a three-dimensional hyperbolic saddle point with a two-dimensional unstable manifold, which locally is a plane through $P_{1}$ generated by the eigenvectors $v_{2}$ and $v_{3}$. There is also a one-dimensional stable manifold which locally is a straight line spanned by the eigenvector $v_{1}$. Trajectories defined by (2.11*a*–*c*) that leave $P_{1}$ must do so via the two-dimensional unstable manifold at $P_{1}$. We therefore compute asymptotic expansions of all solutions of (2.11*a*–*c*) in a neighbourhood of $P_{1}$ that lie on the unstable manifold. Requiring that n∈(0,1) and p<0, so that solutions leaving $P_{1}$ remain in $D_{1}$, we obtain the following result.

**Lemma 3.1.**

*Fix* c>0. *For any* α≥0, *the system* (2.11*a*–*c*) *has a unique solution such that, as* y→−∞,

3.3 $$\begin{array}{rl} & n(y)=1-{\text{e}}^{\lambda_{2}y}+O({\text{e}}^{(\lambda_{2}+\mu )y}),\\ & p(y)=-\lambda_{2}\;{\text{e}}^{\lambda_{2}y}+O({\text{e}}^{(\lambda_{2}+\mu )y})\\ \text{and}\;\; & m(y)=\alpha \;{\text{e}}^{\lambda_{3}y}+O({\text{e}}^{(\lambda_{3}+\mu )y}), \end{array}\}$$

*where* $\lambda_{2}$ *and* $\lambda_{3}$ *are given by* (3.2) *and* $\mu =\min(\lambda_{2},\lambda_{3})>0.$

**Remark 3.2.**

**Figure 1.**
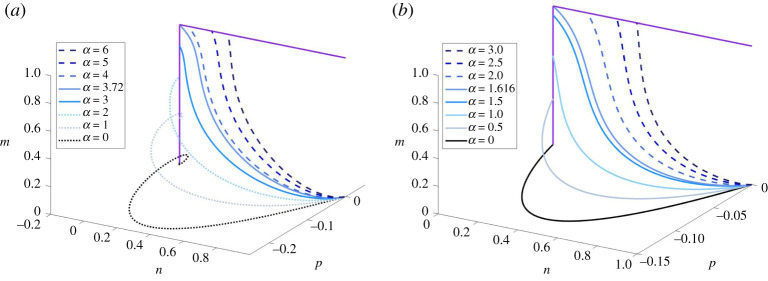
Solutions of (2.11*a*–*c*) subject to the asymptotic conditions (3.3) for different values of the shooting parameter α, κ=1 and c=1 (*a*) or c=2 (*b*). The purple lines are the two continua of steady states of the system (2.11*a*–*c*), given by $\left(0,0,\bar{m}\right)$, $\bar{m}\in [0,1]$, and $\left(\bar{n},0,1\right)$, $\bar{n}\in [0,1]$, respectively. Since $\left(n,m)=(\bar{n},1\right)$, $\bar{n}\in (0,1]$, are not spatially homogeneous steady states of (1.3), the dashed curves represent solutions that are not TWS of system (1.3). The dotted curves represent physically unrealistic solutions for which the n-component becomes negative. The values m and n attain at infinity appear to increase monotonically (between 0 and 1) with α. (Online version in colour.)

The free parameter, α, arises because the form taken by the unstable manifold at $P_{1}$ does not impose any condition on m. In a sense, the choice of α is a choice of how fast m increases from 0 and, accordingly, α will influence the value that m attains at y=+∞. We illustrate this in figure 1 and present some corresponding travelling-wave profiles in electronic supplementary material, S2. In addition, by remark 2.3, it is clear that α=0 is the unique value of the shooting parameter such that the solution of (2.11*a*–*c*) that satisfies (3.3) stays in a region where n∈(0,1), p<0 and m=0 and satisfies the asymptotic conditions (2.10) for $\bar{m}=0$.

Now, the idea is to view solutions of (2.11*a*–*c*) that satisfy (3.3) as functions of α, which we define to be our shooting parameter, and c, which is the wave speed. In particular, we denote by $\left(n_{\alpha ,c},p_{\alpha ,c},m_{\alpha ,c}\right)$ the unique solution of (2.11*a*–*c*) satisfying (3.3). Our first result, which can be proved following the approach in [5], is the following:

**Lemma 3.3.**

*If the solution* $\left(n_{\alpha ,c},p_{\alpha ,c},m_{\alpha ,c}\right)$ *is defined on some interval* $J:=(-\infty ,y_{0})$, *with* $y_{0}\in R$, *and satisfies* $n_{\alpha ,c}(y)>0$ *for all* y∈J, *then* $(n_{\alpha ,c}(y),p_{\alpha ,c}(y),m_{\alpha ,c}(y))\in D_{1}$ *for all* y∈J.

Given lemma 3.3, we introduce the following variable for any α>0 and c>0:

3.4 $$T(\alpha ,c):=\sup\left\{y_{0}\in R|n_{\alpha ,c}(y)>0\;\text{for all}\;y<y_{0}\right\}\in R\cup \{+\infty \}.$$

Then, lemma 3.3 implies that only one of the following holds:

— T(α,c)<+∞, so $n_{\alpha ,c}(T(\alpha ,c))=0$ and $p_{\alpha ,c}(T(\alpha ,c))<0$. In this case, $n_{\alpha ,c}(y)$ becomes negative for some y>T(α,c) and $\left(n_{\alpha ,c},p_{\alpha ,c},m_{\alpha ,c}\right)$ does not represent a valid TWS; we disregard these values of the shooting parameter α.
— T(α,c)=+∞, which means that we have a global solution which stays in $D_{1}$ for all y∈R. We are interested in finding TWS for these values of α.

**Remark 3.4.**

Given $\bar{m}\in (0,1)$, lemma 3.3 provides a condition under which solutions of (2.11*a*–*c*) that satisfy (3.3) remain in $D_{1}$, but not necessarily in $D_{\bar{m}}\subset D_{1}$. In particular, even if $n_{\alpha ,c}(y)>0$ for all y∈J, a solution can leave $D_{\bar{m}}$. In that case, for a solution of (2.11*a*–*c*) that satisfies (3.3) to converge to $\left(0,0,\bar{m}\right)$ as y→+∞, we must have n(y)<0 for some values of y (since m is increasing for positive n). Therefore, searching for solutions that satisfy T(α,c)=+∞ is a necessary condition for the existence of physically realistic TWS that converge to $\left(0,0,\bar{m}\right)$ as y→+∞, but not a sufficient one (m may not attain the value $\bar{m}$ for positive n).

### (b) Monotonicity of solutions with respect to the shooting parameter

A key component of our analysis is that solutions of system (2.11*a*–*c*) satisfying (3.3) are monotonic functions of the shooting parameter, α, provided n>0. This result, which can be proved following the approach in [5], can be formulated as follows:

**Lemma 3.5.**

*Fix* c>0. *If* $\alpha_{2}>\alpha_{1}>0$, *then* $T(\alpha_{2},c)\geq T(\alpha_{1},c)$ *and the solutions of* (2.11*a*–*c*) *defined by* (3.3) *satisfy*

3.5 $$n_{\alpha_{2},c}(y)>n_{\alpha_{1},c}(y),\;m_{\alpha_{2},c}(y)>m_{\alpha_{1},c}(y),$$

*for all* $y\in (-\infty ,T(\alpha_{1},c))$.

Lemma 3.5 shows that, for fixed c>0, T(α,c) is an increasing function of α. Since we seek TWS that satisfy T(α,c)=+∞, we define the following critical value of α, which depends on c:

3.6 $$\alpha_{0}(c):=\inf\left\{\alpha >0|T(\alpha ,c)=+\infty \right\}\in [0,+\infty )\cup \{+\infty \}.$$

We then characterize $\alpha_{0}$ as a function of c in the following lemma, whose proof follows similarly to that of lemma 2.7 in [5]:

**Lemma 3.6.**

*If* c≥2, *then* $\alpha_{0}(c)=0$. *If* 0<c<2, *then* $0<\alpha_{0}(c)<+\infty$.

Lemma 3.6 ensures that for all c>0 there exist some values of α for which T(α,c)=+∞, and thus for all c>0 there exist some TWS. We still need to elucidate the behaviour of solutions at infinity to determine which TWS exist for each c>0.

**Remark 3.7.**

The proof of lemma 3.6 relies on showing that, for any 0<c<2, we can choose α(c)>0 sufficiently large such that there exists a solution of system (2.11*a*–*c*) that satisfies (3.3), remains in region $D_{1}$ and converges to $\left(\bar{n},0,1\right)$ as y→+∞, with $\bar{n}\in (0,1)$. Such solutions are not TWS, but their existence will be crucial in proving the existence of the TWS we seek.

### (c) Behaviour of solutions at infinity

By lemma 3.6, we know that, for any c>0, there exist solutions of system (2.11*a*–*c*) that satisfy (3.3) and remain in region $D_{1}$ for all y∈R. It remains to characterize the behaviour of these solutions as y→+∞, and, in so doing, to establish whether they are TWS. Denoting the limits of components of the solution at infinity as

$$n_{\infty }(\alpha ,c):=\lim_{y\to +\infty }n_{\alpha ,c}(y),\;m_{\infty }(\alpha ,c):=\lim_{y\to +\infty }m_{\alpha ,c}(y),\;p_{\infty }(\alpha ,c):=\lim_{y\to +\infty }p_{\alpha ,c}(y),$$

we introduce the following lemma, which can be proved following the approach in [5].

**Lemma 3.8.**

*If* T(α,c)=+∞, *then the following limits exist*:

3.7 $$n_{\infty }(\alpha ,c)\in [0,1),\;m_{\infty }(\alpha ,c)\in [0,1]\;\text{and}\;p_{\infty }(\alpha ,c)=0.$$

*Moreover, if* $m_{\infty }(\alpha ,c)\in [0,1)$, *then* $n_{\infty }(\alpha ,c)=0$, *and, if* $n_{\infty }(\alpha ,c)\in (0,1)$, *then* $m_{\infty }(\alpha ,c)=1$.

Lemma 3.8 defines the possible limits of solutions $\left(n_{\alpha ,c},p_{\alpha ,c},m_{\alpha ,c}\right)$ of (2.11*a*–*c*) that satisfy (3.3) and remain in region $D_{1}$. We must now determine for which values of c>0 we can find α(c)>0 such that the corresponding solution $\left(n_{\alpha ,c},p_{\alpha ,c},m_{\alpha ,c}\right)$:

(i) remains in $D_{1}$ and converges to (0,0,1) as y→+∞, or,
(i) for each $\bar{m}\in (0,1)$, remains in $D_{\bar{m}}$ and converges to $\left(0,0,\bar{m}\right)$ as y→+∞.

We consider these cases separately in the two sections that follow.

### (d) Solutions converging to the equilibrium point (0,0,1)

In this section, we show that, for each c>0, there exists a unique value of α>0 such that the solution $\left(n_{\alpha ,c},p_{\alpha ,c},m_{\alpha ,c}\right)$ of system (2.11*a*–*c*) satisfying (3.3) remains in region $D_{1}$ and converges to the equilibrium point $P_{2}:=(0,0,1)$ as y→+∞. This then allows us to draw conclusions on the existence and uniqueness of TWS that satisfy the asymptotic conditions (2.2).

By remark 3.7, we have that, for any c>0, there exists α(c)>0 sufficiently large such that the solution of system (2.11*a*–*c*) satisfying (3.3) remains in region $D_{1}$ and satisfies $m_{\infty }=1$. We can therefore define

3.8 $$\alpha_{1}(c):=\inf\left\{\alpha >\alpha_{0}(c)|m_{\infty }(\alpha ,c)=1\right\}\in [0,+\infty )\cup \{+\infty \},$$

and prove the following result:

**Lemma 3.9.**

*For any* c>0, *we have* $0<\alpha_{1}(c)<+\infty$.

**Proof.**

Fix c>0. By remark 3.7, we know that there exists α=α(c)<+∞ large enough such that $m_{\infty }(\alpha ,c)=1$ and, hence, $\alpha_{1}(c)<+\infty$. If 0<c<2, then we know by lemma 3.6 that $\alpha_{0}(c)>0$ and, therefore, by the definition of $\alpha_{1}(c)$, we must have $\alpha_{1}(c)\geq \alpha_{0}(c)>0$.

If c≥2, then suppose, for a contradiction, that $\alpha_{1}(c)=0$. A linear stability analysis about the equilibrium point $\left(0,0,{\bar{m}}_{1}\right)$ with ${\bar{m}}_{1}\in [0,1)$ shows that it is non-hyperbolic with two negative eigenvalues $\lambda_{1}$, $\lambda_{2}$ and one zero eigenvalue $\lambda_{3}$,

3.9 $$\lambda_{1,2}=\frac{-c\pm \sqrt{c^{2}-4(1-{\bar{m}}_{1})}}{2},\;\lambda_{3}=0.$$

Therefore, by the Centre Manifold Theorem, in a small, open neighbourhood of $\left(0,0,{\bar{m}}_{1}\right)$ with ${\bar{m}}_{1}\in [0,1)$, there exists a two-dimensional stable manifold spanned by the eigenvectors $v_{1,2}$ corresponding to $\lambda_{1,2}$. In this neighbourhood, there also exists a one-dimensional centre manifold spanned by $v_{3}={\left(0,0,1\right)}^{⊤}$, which comprises the family of equilibria $\left(0,0,{\bar{m}}_{2}\right)$ with ${\bar{m}}_{2}$ sufficiently close to ${\bar{m}}_{1}$. Therefore, for fixed 0<ϵ<1, we can find a neighbourhood Ω of (0,0,0) that is foliated by two-dimensional stable leaves over a one-dimensional centre manifold, composed of points of the form $\left(0,0,\bar{m}\right)$ with $0\leq \bar{m}<\epsilon$. Then, any solution that enters Ω converges to $\left(0,0,\bar{m}\right)$ as y→+∞ for some $\bar{m}$ that satisfies $0\leq \bar{m}<\epsilon$.

Now, by remark 3.2, $\alpha_{1}(c)=0$ implies that $\left(n_{\infty }(\alpha_{1}(c),c),p_{\infty }(\alpha_{1}(c),c),m_{\infty }(\alpha_{1}(c),c))=(0,0,0\right)$. Thus, we can find $\bar{y}\in R$ large enough such that $(n_{\alpha_{1}(c),c}(y),p_{\alpha_{1}(c),c}(y),m_{\alpha_{1}(c),c}(y))\in \Omega$ for all $y\geq \bar{y}$. By continuity of solutions with respect to α, we can find δ>0 such that $(n_{\alpha ,c}(\bar{y}),p_{\alpha ,c}(\bar{y}),m_{\alpha ,c}(\bar{y}))\in \Omega$ for any 0<α<δ. This implies that, for any such α, $\left(n_{\alpha ,c}(y),p_{\alpha ,c}(y),m_{\alpha ,c}(y)\right)$ converges to $(0,0,\bar{m})\in \Omega$ as y→+∞. Since $0\leq \bar{m}<\epsilon$ by our choice of Ω, there exists 0<α<δ such that $0\leq m_{\infty }(\alpha ,c)<\epsilon$. However, since $\alpha_{1}(c)=0$, we must have $m_{\infty }(\alpha ,c)=1$ for all α>0 and we have reached the desired contradiction.

Lemma 3.9 ensures that, for any c>0 and $\alpha \geq \alpha_{1}(c)$, the solution $\left(n_{\alpha ,c},p_{\alpha ,c},m_{\alpha ,c}\right)$ of (2.11*a*–*c*), subject to the asymptotic conditions (3.3), stays in region $D_{1}$ and satisfies $\left(n_{\infty }(\alpha ,c),p_{\infty }(\alpha ,c),m_{\infty }(\alpha ,c))=(n_{\infty }(\alpha ,c),0,1\right)$, where $n_{\infty }(\alpha ,c)\in [0,1)$. We would now like to show that, for any c>0, there exists a unique $\alpha \geq \alpha_{1}(c)$ such that $n_{\infty }(\alpha ,c)=0$.

For the rest of this section, we suppose that $\alpha \geq \alpha_{1}(c)$. A linear stability analysis at the equilibrium point $P_{2}$ shows that $P_{2}$ is non-hyperbolic, with one negative eigenvalue and two zero eigenvalues. Therefore, at $P_{2}$, we have a one-dimensional stable manifold, $W_{S}\subset R^{3}$, generated by the eigenvector $v_{1}={\left(1/c,1,0\right)}^{⊤}$ associated with $\lambda_{1}=-c$. We also have a two-dimensional centre manifold, $W_{C}\subset R^{3}$, which is tangent at $P_{2}$ to the subspace spanned by the eigenvectors $v_{2}={\left(1,0,0\right)}^{⊤}$ and $v_{3}={\left(0,0,1\right)}^{⊤}$ associated with $\lambda_{2}=\lambda_{3}=0$. Solutions of (2.11*a*–*c*) that satisfy (3.3) and remain in a small enough neighbourhood of $P_{2}$ for all sufficiently large y>0 converge to $W_{C}$. Therefore, in order to study the dynamics around $P_{2}$, we perform a nonlinear local stability analysis. We begin by transforming system (2.11*a*–*c*) into normal form by introducing the following variables:

3.10 $$\tilde{n}(y)=n(y)+\frac{p(y)}{c},\;\tilde{p}(y)=p(y),\;\tilde{m}(y)=1-m(y),$$

which satisfy the following system:

3.11a $$\begin{array}{rl} & \frac{\text{d}\tilde{n}}{\text{d}y}=-\frac{1}{c}\tilde{m}\left(\tilde{n}-\frac{\tilde{p}}{c}\right)\left(1-\tilde{n}+\frac{\tilde{p}}{c}\right), \end{array}$$

3.11b $$\begin{array}{rl} & \frac{\text{d}\tilde{p}}{\text{d}y}=-c\tilde{p}-\tilde{m}\left(\tilde{n}-\frac{\tilde{p}}{c}\right)\left(1-\tilde{n}+\frac{\tilde{p}}{c}\right) \end{array}$$

3.11c $$\begin{array}{rl} \text{and}\;\; & \frac{\text{d}\tilde{m}}{\text{d}y}=-\frac{\kappa }{c}\tilde{m}(1-\tilde{m})\left(\tilde{n}-\frac{\tilde{p}}{c}\right). \end{array}$$

Then, we know that, in a neighbourhood of the origin, the centre manifold can be described by a function $P(\tilde{n},\tilde{m})$ such that $(\tilde{n},\tilde{p},\tilde{m})\in W_{C}$ if and only if $\tilde{p}=P(\tilde{n},\tilde{m})$, where

3.12 $$P(\tilde{n},\tilde{m})=-\frac{1}{c}\tilde{n}\tilde{m}(1+O(|\tilde{n}|+|\tilde{m}|)).$$

Using this expression for the centre manifold in a neighbourhood of the origin, we must now prove that there is a solution of system (3.11*a*–*c*) converging to the centre manifold $W_{C}$ that converges to the origin as y→+∞. We are interested in solutions (n,p,m) of (2.11*a*–*c*) that satisfy (3.3) and remain in region $D_{1}$ for all y∈R. Equivalently, we seek solutions $\left(\tilde{n},\tilde{p},\tilde{m}\right)$ of (3.11*a*–*c*) that satisfy (3.3) and lie on a manifold $W_{C}^{+}\subset W_{C}$, where

3.13 $$W_{C}^{+}=\{(\tilde{n},\tilde{p},\tilde{m})\in W_{C}|\tilde{n},\tilde{m}>0\}.$$

The following lemma characterizes such solutions that converge to the origin as y→+∞ (the proof corresponds, with minor modifications, to that of lemma 2.12 in [5]).

**Lemma 3.10.**

*Up to translations in the variable* y, *there exists a unique solution of* (3.11*a*–*c*) *that satisfies the asymptotic conditions* (3.3), *lies on the centre manifold* $W_{C}^{+}$ *and whose components converge to zero as* y→+∞, *such that*

3.14 $$\tilde{n}(y)=\frac{c}{\kappa y}+O\left(\frac{1}{y^{2}}\right)\;\text{and}\;\tilde{m}(y)=\frac{c}{y}+O\left(\frac{1}{y^{2}}\right).$$

Lemma 3.10 establishes the existence of at least one solution of (2.11*a*–*c*) that satisfies (3.3), stays in region $D_{1}$ and converges to (1,0,0) as y→+∞. Furthermore, this solution is uniquely determined on the centre manifold $W_{C}^{+}$. Given that any solution of (2.11*a*–*c*) that satisfies (3.3), stays in region $D_{1}$ and converges to (0,0,1) as y→+∞ must do so via $W_{C}^{+}$ and, given the monotonicity result of lemma 3.5, it is easy to prove the following lemma as in [5].

**Lemma 3.11.**

*Given any* c>0, *there exists at most one value of* $\alpha \geq \alpha_{1}(c)$ *of the shooting parameter such that the solution* $\left(n_{\alpha ,c}(y),p_{\alpha ,c}(y),m_{\alpha ,c}(y)\right)$ *of* (2.11*a*–*c*) *satisfying the asymptotic properties in lemma 3.1 converges to* $P_{2}=(0,0,1)$ *as* y→+∞.

Exploiting the continuity of solutions with respect to the shooting parameter, α, we can extend lemma 3.11 to determine the unique value of α, given c>0, for which the solution $\left(n_{\alpha ,c}(y),p_{\alpha ,c}(y),m_{\alpha ,c}(y)\right)$ of (2.11*a*–*c*) that satisfies (3.3) converges to (0,0,1) as y→+∞. For the proof of the following result, we refer the reader to the proof of lemma 2.14 in [5].

**Lemma 3.12.**

*Given any* c>0, *if* $\alpha =\alpha_{1}(c)$, *then the solution* $\left(n_{\alpha ,c}(y),p_{\alpha ,c}(y),m_{\alpha ,c}(y)\right)$ *of* (2.11*a*–*c*) *satisfying the asymptotic properties in lemma 3.1 converges to* $P_{2}=(0,0,1)$ *as* y→+∞.

Using lemma 3.12 and reversing the change of variables (2.6), it is straightforward to construct a unique (up to translation) solution (N(ξ),M(ξ))=(n(Φ(ξ)),m(Φ(ξ))) of system (2.1*a*,b) that satisfies the asymptotic conditions (2.2). This leads to our first main result, which can be proved following the approach in [5]:

**Theorem 3.13.**

*Fix* κ>0. *For any* c>0, *system* (1.3) *has a smooth weak TWS* (N,M;c) *connecting* (1,0) *and* (0,1). *This solution is unique (up to translation), and* N *and* M *are monotonically strictly decreasing and increasing functions of* x−ct=ξ∈R∪{−∞,+∞}, *respectively*.

### (e) Solutions converging to the equilibrium point $\left(0,0,\bar{m}\right)$ with $\bar{m}\in [0,1)$

In this section, we consider solutions of system (2.11*a*–*c*) subject to (3.3) that stay in region $D_{\bar{m}}\subset D_{1}$ and converge to $\left(0,0,\bar{m}\right)$ for $\bar{m}\in (0,1)$ as y→+∞. Using arguments similar to those for the previous case, we can show that, for all $\bar{m}\in [0,1)$, there exists a strictly positive, real-valued wave speed above which the solutions we seek exist and are unique. We will refer to this wave speed as the *minimal wave speed* and we will observe that it depends on κ, the rescaled degradation rate of ECM. In particular, given κ>0, we denote the minimal wave speed by $c_{\kappa }^{*}(\bar{m})$ for each $\bar{m}\in [0,1)$. This will enable us to draw some conclusions on the existence and uniqueness of TWS that satisfy the asymptotic conditions (2.3).

At this stage, we have no information about the possible values of $c_{\kappa }^{*}(\bar{m})$ for $\bar{m}\in (0,1)$ and κ>0. More specifically, given κ>0, we currently have $c_{\kappa }^{*}(\bar{m})\in R_{+}^{*}$ for each $\bar{m}\in (0,1)$. For $\bar{m}=0$, by remark 2.3, it is straightforward to show that $c_{\kappa }^{*}(0)=2$ for all κ>0. To characterize the minimal wave speed for $\bar{m}\in (0,1)$, we begin by proving a non-existence result.

**Lemma 3.14.**

*Fix* κ>0 *and* $\bar{m}\in (0,1)$. *If* $0<c<2\sqrt{1-\bar{m}}$, *then there is no* $\alpha^{\prime }\in [\alpha_{0},+\infty )$ *such that the solution* $\left(n_{\alpha^{\prime },c},p_{\alpha^{\prime },c},m_{\alpha^{\prime },c}\right)$ *of* (2.11*a*–*c*) *that satisfies the asymptotic properties in lemma 3.1 converges to* $\left(0,0,\bar{m}\right)$ *as* y→+∞.

**Proof.**

Fix κ>0 and $\bar{m}\in (0,1)$ and suppose that $0<c<2\sqrt{1-\bar{m}}$. We suppose for a contradiction that there exists $\alpha^{\prime }\in [\alpha_{0},+\infty )$ such that the solution $\left(n_{\alpha^{\prime },c},p_{\alpha^{\prime },c},m_{\alpha^{\prime },c}\right)$ of (2.11*a*–*c*) that satisfies (3.3) converges to $\left(0,0,\bar{m}\right)$ as y→+∞. By the definition of $\alpha_{0}(c)$, this implies that $\left(n_{\alpha^{\prime },c},p_{\alpha^{\prime },c},m_{\alpha^{\prime },c}\right)$ stays in region $D_{1}$ for all y∈R. Now, we can choose ϵ>0 small enough such that $0<c<2\sqrt{\left(1-\bar{m}-\epsilon )(1-\epsilon \right)}$ and we can also find $\bar{y}$ sufficiently large such that $n_{\alpha^{\prime },c}(y)<\epsilon$ and $m_{\alpha^{\prime },c}(y)<\bar{m}+\epsilon$ for all $y\geq \bar{y}$. Solutions of the constant coefficient second-order ODE

$$n^{\prime \prime }+cn^{\prime }+(1-\bar{m}-\epsilon )(1-\epsilon )n=0,$$

$$\text{with}\;\lim_{y\to -\infty }n(y)=1,\;\lim_{y\to +\infty }n(y)=0,\;\lim_{y\to \pm \infty }n^{\prime }(y)=0,$$

have infinitely many zeros in $\left(\bar{y},+\infty \right)$ (since its characteristic equation has complex roots). Since $\left(1-\bar{m}-\epsilon )(1-\epsilon )<(1-m_{\alpha^{\prime },c}(y))(1-n_{\alpha^{\prime },c}(y)\right)$ for all $y\in (\bar{y},+\infty )$, Sturm’s Comparison Theorem implies that $n_{\alpha^{\prime },c}(y)$ must also have infinitely many zeros in $\left(\bar{y},+\infty \right)$. Therefore, $\left(n_{\alpha^{\prime },c},p_{\alpha^{\prime },c},m_{\alpha^{\prime },c}\right)$ exits region $D_{1}$ (and $D_{\bar{m}}$), contradicting the assumption that $\alpha^{\prime }\geq \alpha_{0}(c)$.

Given κ>0 and $\bar{m}\in (0,1)$, if the minimal wave speed, $c_{\kappa }^{*}(\bar{m})$, exists, then lemma 3.14 yields a lower bound for $c_{\kappa }^{*}(\bar{m})$. More specifically, for all κ>0 and $\bar{m}\in (0,1)$, $c_{\kappa }^{*}(\bar{m})\geq 2\sqrt{\left(1-\bar{m}\right)}$.

**Lemma 3.15.**

*Fix* κ>0. *If* c≥2, *then, for all* $\bar{m}\in (0,1)$, *there exists a unique* $\alpha \in (\alpha_{0}(c),\alpha_{1}(c))$ *such that the solution of* (2.11*a*–*c*) *satisfying the asymptotic properties in lemma 3.1 converges to* $\left(0,0,\bar{m}\right)$ *as* y→+∞.

**Proof.**

Fix κ>0 and suppose that c≥2. By lemmas 3.6 and 3.9, we know that $0=\alpha_{0}(c)<\alpha_{1}(c)$. Then, by the definition of $\alpha_{0}(c)$, we have that, for any $\alpha \in (\alpha_{0}(c),\alpha_{1}(c))$, T(α,c)=+∞, and the solution $\left(n_{\alpha ,c},p_{\alpha ,c},m_{\alpha ,c}\right)$ of (2.11*a*–*c*) satisfying (3.3) stays in the region $D_{1}$ for all y∈R by lemma 3.3. Then, by lemma 3.8, we know that, for every $\alpha \in (\alpha_{0}(c),\alpha_{1}(c))$, the limits $n_{\infty }(\alpha ,c)$, $p_{\infty }(\alpha ,c)$, $m_{\infty }(\alpha ,c)$ exist. In addition, by monotonicity of $n_{\alpha ,c}$ and $m_{\alpha ,c}$ with respect to α (see lemma 3.5) and the fact that $\left(n_{\infty }(\alpha_{1}(c),c),m_{\infty }(\alpha_{1}(c),c))=(0,1\right)$ by lemma 3.12, we must have

$$0=n_{\infty }(\alpha_{0}(c),c)\leq n_{\infty }(\alpha ,c)\leq n_{\infty }(\alpha_{1}(c),c)=0$$

and

$$0=m_{\infty }(\alpha_{0}(c),c)\leq m_{\infty }(\alpha ,c)\leq m_{\infty }(\alpha_{1}(c),c)=1.$$

We recall that $\alpha_{0}(c)=0$ and $\alpha_{1}(c)$ are the unique values of the shooting parameter for which the solution of (2.11*a*–*c*) that satisfies (3.3) remains in region $D_{1}$ and converges to (0,0,0) and (0,0,1) as y→+∞, respectively. Therefore, we find that, for every $\alpha \in (\alpha_{0}(c),\alpha_{1}(c))$, the limits for $\left(n_{\alpha ,c},p_{\alpha ,c},m_{\alpha ,c}\right)$ as y→+∞ must satisfy

3.15 $$n_{\infty }(\alpha ,c)=0,\;m_{\infty }(\alpha ,c)\in (0,1)\;\text{and}\;p_{\infty }(\alpha ,c)=0.$$

We will now prove that the mapping $\alpha \mapsto m_{\infty }(\alpha ,c)$ is continuous and strictly increasing on $\left[\alpha_{0}(c),\alpha_{1}(c)\right]$. Choose $\alpha^{\prime }$, $\alpha^{\prime \prime }$ such that $\alpha_{0}(c)<\alpha^{\prime }<\alpha^{\prime \prime }<\alpha_{1}(c)$. Suppose, for a contradiction, that

$$\lim_{y\to +\infty }m_{\alpha^{\prime },c}(y)=m_{\infty }(\alpha^{\prime },c)=\bar{m}=m_{\infty }(\alpha^{\prime \prime },c)=\lim_{y\to +\infty }m_{\alpha^{\prime \prime },c}(y).$$

Irrespective of the asymptotic conditions (3.3), we can solve equation (2.11c) for y∈R and impose $\lim_{y\to +\infty }m(y)=\bar{m}\in (0,1)$ to obtain

3.16 $$m(y)={\left[1+\frac{1-\bar{m}}{\bar{m}}\exp\left(\frac{\kappa }{c}\int_{y}^{+\infty }n(s)\;ds\right)\right]}^{-1}.$$

Any solution for which $\lim_{y\to +\infty }m(y)=\bar{m}\in (0,1)$ must therefore take the form (3.16). Thus, $m_{\alpha^{\prime },c}$ and $m_{\alpha^{\prime \prime },c}$ take the form (3.16), with n replaced by $n_{\alpha^{\prime },c}$ and $n_{\alpha^{\prime \prime },c}$, respectively. Now, by lemma 3.5, we know that $m_{\alpha^{\prime },c}(y)<m_{\alpha^{\prime \prime },c}(y)$ for all y∈R since $\alpha^{\prime }<\alpha^{\prime \prime }$. We therefore have, for any y∈R,

3.17 $$\begin{array}{rl} & {\left[1+\frac{1-\bar{m}}{\bar{m}}\exp\left(\frac{\kappa }{c}\int_{y}^{+\infty }n_{\alpha^{\prime },c}(s)\;ds\right)\right]}^{-1}<{\left[1+\frac{1-\bar{m}}{\bar{m}}\exp\left(\frac{\kappa }{c}\int_{y}^{+\infty }n_{\alpha^{\prime \prime },c}(s)\;ds\right)\right]}^{-1} \end{array}$$

3.18 $$\begin{array}{rl} & \;\Rightarrow \int_{y}^{+\infty }(n_{\alpha^{\prime },c}(s)-n_{\alpha^{\prime \prime },c}(s))\;ds>0. \end{array}$$

Since $(n_{\alpha^{\prime },c}(y)-n_{\alpha^{\prime \prime },c}(y))<0$ for all y∈R by lemma 3.5, the inequality (3.18) cannot hold and we have reached a contradiction. Since we have that $m_{\infty }(\alpha^{\prime },c)\leq m_{\infty }(\alpha^{\prime \prime },c)$, by monotonicity of solutions with respect to α, and that $m_{\infty }(\alpha^{\prime },c)\neq m_{\infty }(\alpha^{\prime \prime },c)$, by the above argument, we conclude that $m_{\infty }(\alpha^{\prime },c)<m_{\infty }(\alpha^{\prime \prime },c)$. This proves that the mapping $\alpha \mapsto m_{\infty }(\alpha ,c)$ is strictly increasing on $\left(\alpha_{0}(c),\alpha_{1}(c)\right)$. Using the fact that $\alpha_{0}(c)$ and $\alpha_{1}(c)$ are, respectively, the unique values of the shooting parameter for which the solution of (2.11*a*–*c*) given by lemma 3.1 converges to (0,0,0) and (0,0,1) as y→+∞, we have that $\alpha \mapsto m_{\infty }(\alpha ,c)$ is strictly increasing on $\left[\alpha_{0}(c),\alpha_{1}(c)\right]$.

We now prove that the mapping $\alpha \mapsto m_{\infty }(\alpha ,c)$ is continuous on $\left[\alpha_{0}(c),\alpha_{1}(c)\right]$. For fixed $\alpha^{\prime }\in [\alpha_{0}(c),\alpha_{1}(c))$, (3.15) implies that $n_{\infty }(\alpha^{\prime },c)=0,p_{\infty }(\alpha^{\prime },c)=0$ and $m_{\infty }(\alpha^{\prime },c)={\bar{m}}_{1}\in [0,1)$. In the proof of lemma 3.9, we performed a linear stability analysis about the equilibrium point $\left(0,0,{\bar{m}}_{1}\right)$ for ${\bar{m}}_{1}\in [0,1)$. We showed that, for fixed ϵ>0, we can find a neighbourhood Ω of $\left(0,0,{\bar{m}}_{1}\right)$ that is foliated by two-dimensional stable leaves over a one-dimensional centre manifold, which comprises equilibria of the form $\left(0,0,\bar{m}\right)$ for $|\bar{m}-{\bar{m}}_{1}|<\epsilon$. Then, any solution that enters Ω converges to $\left(0,0,\bar{m}\right)$ as y→+∞ for some $\bar{m}$ that satisfies $|\bar{m}-{\bar{m}}_{1}|<\epsilon$. Since $\left(n_{\alpha^{\prime },c}(y),p_{\alpha^{\prime },c}(y),m_{\alpha^{\prime },c}(y)\right)$ converges to $\left(0,0,{\bar{m}}_{1}\right)$ as y→+∞, we can find $\bar{y}\in R$ large enough such that $(n_{\alpha^{\prime },c}(y),p_{\alpha^{\prime },c}(y),m_{\alpha^{\prime },c}(y))\in \Omega$ for all $y\geq \bar{y}$. By continuity of solutions with respect to α, we can find δ>0 such that $(n_{\alpha^{\prime \prime },c}(\bar{y}),p_{\alpha^{\prime \prime },c}(\bar{y}),m_{\alpha^{\prime \prime },c}(\bar{y}))\in \Omega$ for any $\alpha^{\prime \prime }\in [\alpha_{0}(c),\alpha_{1}(c))$ such that $|\alpha^{\prime }-\alpha^{\prime \prime }|<\delta$. This implies that, for any such $\alpha^{\prime \prime }$, $\left(n_{\alpha^{\prime \prime },c}(y),p_{\alpha^{\prime \prime },c}(y),m_{\alpha^{\prime \prime },c}(y)\right)$ converges to $(0,0,{\bar{m}}_{2})\in \Omega$ as y→+∞, for some ${\bar{m}}_{2}\neq {\bar{m}}_{1}$ (since $m_{\infty }(\alpha ,c)$ is strictly increasing with α). By our choice of Ω, $|{\bar{m}}_{2}-{\bar{m}}_{1}|<\epsilon$, i.e. $|m_{\infty }(\alpha^{\prime \prime },c)-m_{\infty }(\alpha^{\prime },c)|<\epsilon$ for any $\alpha^{\prime \prime }\in [\alpha_{0}(c),\alpha_{1}(c))$ such that $|\alpha^{\prime }-\alpha^{\prime \prime }|<\delta$. This proves continuity of the mapping $\alpha \mapsto m_{\infty }(\alpha ,c)$ on $\left[\alpha_{0}(c),\alpha_{1}(c)\right)$.

We finally show continuity at $\alpha_{1}(c)$. We fix ϵ>0 and note that, since $m_{\infty }(\alpha_{1}(c),c)=1$, we can find $\bar{y}\in R$ large enough such that $|m_{\alpha_{1}(c),c}(y)-1|<\epsilon /2$ for all $y\geq \bar{y}$. By continuity of solutions with respect to α, we can find δ>0 such that $|m_{\alpha^{\prime },c}(\bar{y})-m_{\alpha_{1}(c),c}(\bar{y})|<\epsilon /2$ for any $\alpha^{\prime }\in [\alpha_{0}(c),\alpha_{1}(c)]$ satisfying $|\alpha^{\prime }-\alpha_{1}(c)|<\delta$, i.e. for any $\alpha^{\prime }\in (\alpha_{1}(c)-\delta ,\alpha_{1}(c)]$. Therefore, we have that $|m_{\alpha^{\prime },c}(\bar{y})-1|<\epsilon$ for any $\alpha^{\prime }\in (\alpha_{1}(c)-\delta ,\alpha_{1}(c)]$. Moreover, for any $\alpha^{\prime }\in (\alpha_{1}(c)-\delta ,\alpha_{1}(c)]$, the function $m_{\alpha^{\prime },c}(y)$ is strictly increasing for all y∈R and bounded above by 1, so $m_{\infty }(\alpha^{\prime },c)\in (m_{\alpha^{\prime },c}(\bar{y}),1]$. In particular, for any $\alpha^{\prime }\in (\alpha_{1}(c)-\delta ,\alpha_{1}(c)]$, we have $|m_{\infty }(\alpha^{\prime },c)-1|=|m_{\infty }(\alpha^{\prime },c)-m_{\infty }(\alpha_{1}(c),c)|<\epsilon$. This proves continuity of the mapping $\alpha \mapsto m_{\infty }(\alpha ,c)$ at $\alpha_{1}(c)$.

We have now shown that the mapping $\alpha \mapsto m_{\infty }(\alpha ,c)$ is strictly increasing and continuous on $\left[\alpha_{0}(c),\alpha_{1}(c)\right]$. Since $m_{\infty }(\alpha_{0}(c),c)=0$ and $m_{\infty }(\alpha_{1}(c),c)=1$, application of the Intermediate Value Theorem enables us to conclude that, for any $\bar{m}\in (0,1)$, there exists a unique $\alpha \in (\alpha_{0}(c),\alpha_{1}(c))$ such that $m_{\infty }(\alpha ,c)=\bar{m}$.

**Remark 3.16.**

Using a similar proof to the above, we can generalize lemma 3.15 to obtain the following result. Given κ,c>0, suppose that there exists a unique value of the shooting parameter, $\alpha \in [\alpha_{0}(c),\alpha_{1}(c))$, such that the solution $\left(n_{\alpha ,c},p_{\alpha ,c},m_{\alpha ,c}\right)$ of (2.11*a*–*c*) satisfies (3.3) and converges to $\left(0,0,{\bar{m}}_{1}\right)$ as y→+∞ for some ${\bar{m}}_{1}\in [0,1)$. Then, for all ${\bar{m}}_{2}\in ({\bar{m}}_{1},1)$, there exists a unique value of the shooting parameter, $\alpha^{\prime }\in (\alpha ,\alpha_{1}(c))$, for which the solution of (2.11*a*–*c*) that satisfies (3.3) stays in $D_{{\bar{m}}_{2}}$ and converges to $\left(0,0,{\bar{m}}_{2}\right)$ as y→+∞.

Lemma 3.15 implies that, for all $\bar{m}\in (0,1)$, the minimal wave speed, $c_{\kappa }^{*}(\bar{m})$, exists and is bounded above by 2. Then, given $\bar{m}\in [0,1)$, for any $c\geq c_{\kappa }^{*}(\bar{m})$, we can define

3.19 $$\alpha_{\bar{m}}(c):=\left\{\alpha \geq \alpha_{0}(c)|m_{\infty }(\alpha ,c)=\bar{m}\right\}\in [\alpha_{0}(c),\alpha_{1}(c)).$$

We now improve the upper bound on $c_{\kappa }^{*}(\bar{m})$ for $\bar{m}\in (0,1)$ by formulating a conjecture. We consider the following generalized Fisher–KPP equation with reaction term, g, of Fisher–KPP type,

3.20 $$\begin{array}{rl} & n^{\prime \prime }+cn^{\prime }+g(n)=0\\ \text{with}\; & \;\lim_{y\to -\infty }n(y)=1,\;\lim_{y\to +\infty }n(y)=0,\;\lim_{y\to \pm \infty }n^{\prime }(y)=0. \end{array}\}$$

One typically seeks TWS such that n is monotonically decreasing, in which case we can invert n(y) to obtain a function Y(n), n∈[0,1]. Considering the new variable $P(n):=n^{\prime }(Y(n))$, we obtain the following first-order boundary value problem (BVP):

3.21 $$\begin{array}{rl} & P^{\prime }=-c-\frac{g(n)}{P}\\ \text{and}\;\; & P(0)=0, \end{array}\}$$

subject to P(1)=0, P(n)<0 ∀ n∈(0,1). Studying TWS of (3.20) and solutions of (3.21), subject to their respective asymptotic and boundary conditions, is equivalent [22]. Moreover, it is known that if $g^{\prime \prime }(n)<0\;\forall \;n\in [0,1]$, then (3.21) subject to P(1)=0, P(n)<0 ∀ n∈(0,1) has a unique solution if $c\geq 2\sqrt{g^{\prime }(0)}$ [40–42]. Therefore, TWS of (3.20) exist and are unique if $c\geq 2\sqrt{g^{\prime }(0)}$.

Returning to our original problem, by introducing $P(n):=n^{\prime }(Y(n))$ and M(n):=m(Y(n)), we view the system (2.11*a*–*c*) subject to the conditions (2.3) as the following BVP:

3.22 $$\begin{array}{rl} & P^{\prime }=-c-\frac{\left(1-n)n(1-M(n)\right)}{P},\\ & M^{\prime }=\frac{\kappa }{c}\frac{M(1-M)N}{P}\\ \text{and}\;\; & P(0)=0,M(0)=\bar{m}, \end{array}\}$$

subject to the additional conditions

3.23 $$P(n)<0,\;0<M(n)<\bar{m}\;\forall \;n\in (0,1),\;P(1)=M(1)=0.$$

In electronic supplementary material, S1, we show that g(n)=(1−n)n(1−M(n)) is of Fisher–KPP type for $0\leq M\leq \bar{m}<1$ and that $g^{\prime \prime }(0)<0$ if $\kappa \leq \kappa^{*}(\bar{m})$, where

3.24 $$\kappa^{*}(\bar{m}):=\frac{1-\bar{m}}{\bar{m}}\;\forall \;\bar{m}\in (0,1).$$

We conjecture that, if $\kappa \leq \kappa^{*}(\bar{m})$, then $g^{\prime \prime }(n)<0\;\forall \;n\in [0,1]$. By the preceding result for the generalized Fisher–KPP equation, this would imply that, given $0<\kappa \leq \kappa^{*}(\bar{m})$, the system (2.11*a*–*c*) subject to the conditions (2.3) has unique TWS for $c\geq 2\sqrt{g^{\prime }(0)}=2\sqrt{1-\bar{m}}$. Now, given κ>0, we let $m^{*}(\kappa ):=1/(\kappa +1)$. Noting that $0<\kappa \leq \kappa^{*}(\bar{m})$ if and only if $0<\bar{m}\leq m^{*}(\kappa )$, we formulate the following conjecture.

**Conjecture 3.17.**

Fix κ>0 and $\bar{m}\in (0,m^{*}(\kappa )]$. Given $c\geq 2\sqrt{1-\bar{m}}$, there exists a unique $\alpha^{\prime }\in [\alpha_{0}(c),\alpha_{1}(c))$ such that the solution $\left(n_{\alpha^{\prime },c},p_{\alpha^{\prime },c},m_{\alpha^{\prime },c}\right)$ of (2.11*a*–*c*) that satisfies the asymptotic properties in lemma 3.1 converges to $\left(0,0,\bar{m}\right)$ as y→+∞. In particular, if $c=2\sqrt{1-\bar{m}}$, then $\alpha^{\prime }=\;\alpha_{0}(c)$.

Conjecture 3.17 implies that, given κ>0, there are values of $\bar{m}\in (0,1)$ such that the solutions of (2.11*a*–*c*) that satisfy the asymptotic conditions (2.3) behave similarly to solutions of a generalized Fisher–KPP equation with reaction term $g(n)=(1-n)n(1-\bar{m})$. In particular, the minimal wave speed for these TWS is defined similarly to that of a generalized Fisher–KPP equation, i.e. it is the smallest value of c>0 such that $\left(0,0,\bar{m}\right)$ is a stable node, and not a stable spiral, for system (2.11*a*–*c*). In addition, using lemmas 3.14 and 3.15 and conjecture 3.17, we make the hypothesis that, if $\bar{m}\in (m^{*}(\kappa ),1)$ or, equivalently, if $\kappa >\kappa^{*}(\bar{m})$, then the minimal wave speed for TWS that converge to $\left(0,0,\bar{m}\right)$ as y→+∞ should satisfy $c_{\kappa }^{*}(\bar{m})\in (2\sqrt{1-\bar{m}},2)$. In other words, in these cases, we expect that there is another mechanism that can lead to n(y)<0, y∈R, even if $\left(0,0,\bar{m}\right)$ is a stable node for the system (2.11*a*–*c*).

**Figure 2.**
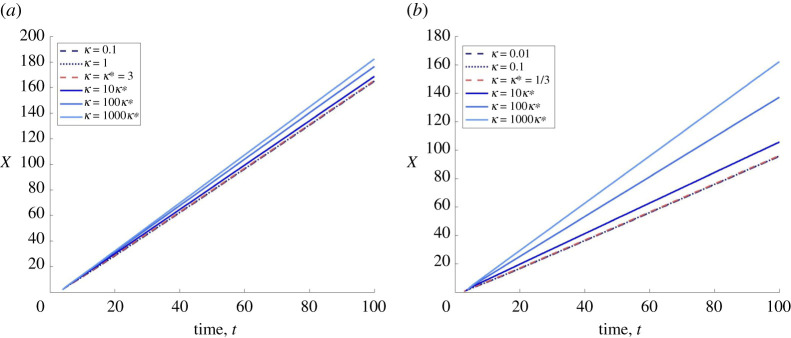
We numerically solve system (1.3) on the one-dimensional spatial domain, x∈X:=[0,200], and impose the initial conditions (4.1) with $\bar{M}=0.25$ (*a*) and $\bar{M}=0.75$ (*b*). Each plot represents X(t) such that N(X(t),t)=0.5 for t∈(0,100] when $\kappa <\kappa^{*}$, with $\kappa^{*}$ defined by (3.24), and when $\kappa \in \{\kappa^{*},10\kappa^{*},100\kappa^{*},1000\kappa^{*}\}$. We see that the front travels with a constant propagation speed that increases monotonically with κ. (Online version in colour.)

The preceding hypothesis and conjecture 3.17 are supported by numerical simulations of the PDE system (1.3) and ODE system (2.11*a*–*c*). In figure 2, we show that solutions of system (1.3) subject to the initial conditions (4.1) with $\bar{M}\in [0,1)$ evolve into travelling waves with constant propagation speed (see electronic supplementary material, S2 for corresponding travelling-wave profiles). We observe that, for $0<\kappa \leq \kappa^{*}(\bar{M})$, this speed is independent of κ, and, calculating the slopes of these lines, we find that it is approximately equal to $2\sqrt{1-\bar{M}}$. Additionally, when $\kappa >\kappa^{*}(\bar{M})$, we observe that the wave speed selected by the PDE increases with κ. We also solved numerically the system (2.11*a*–*c*), subject to the asymptotic conditions (3.3), for the same values of $\kappa >\kappa^{*}(\bar{M})$ and the respective values of the propagation speed estimated using the solutions of the PDE system (results not shown). We observed that, given $\kappa >\kappa^{*}(\bar{M})$, the wave speed selected by the PDE appears to correspond to the smallest wave speed such that the solution (n,p,m) of the system (2.11*a*–*c*), subject to (3.3), satisfies n(y)>0 ∀ y∈R and converges to $\left(0,0,\bar{m}\right)$, $\bar{m}=\bar{M}$.

Now, suppose conjecture 3.17 is true. Then, given κ>0, for each $\bar{m}\in [0,m^{*}(\kappa )]$ and $c\geq 2\sqrt{1-\bar{m}}$, $\alpha_{\bar{m}}(c)$ as defined by (3.19) exists and is the unique $\alpha^{\prime }$ mentioned in the statement of conjecture 3.17. Using remark 3.16 and conjecture 3.17, the subsequent result follows naturally (we omit the proof for brevity).

**Lemma 3.18.**

*Suppose conjecture 3.17 is true and fix* κ>0. *If* $c\geq 2\sqrt{1-m^{*}(\kappa )}$, *then, for all* $\bar{m}\in (m^{*}(\kappa ),1)$, *there exists a unique* $\alpha \in (\alpha_{m^{*}(\kappa )}(c),\alpha_{1}(c))$ *such that the solution of* (2.11*a*–*c*) *that satisfies the asymptotic properties in lemma 3.1 converges to* $\left(0,0,\bar{m}\right)$ *as* y→+∞.

This lemma allows us to obtain a sharper upper bound on the minimal wave speed for solutions of (2.11*a*–*c*) subject to (3.3) that converge to $\left(0,0,\bar{m}\right)$ as y→+∞, where $\bar{m}\in (m^{*}(\kappa ),1)$. We now summarize what we can conclude about the minimal wave speed $c_{\kappa }^{*}(\bar{m})$.

**Lemma 3.19.**

*Suppose conjecture 3.17 is true. Given* κ>0, *the minimal wave speed* $c_{\kappa }^{*}(\bar{m})$ *is a monotonically decreasing function on* [0,1), *such that*

3.25 $$c_{\kappa }^{*}(\bar{m})\left\{ \begin{array}{ll} =2\sqrt{1-\bar{m}} & \text{if}\;\bar{m}\in [0,m^{*}(\kappa )],\\ \in \left[2\sqrt{1-\bar{m}},2\sqrt{1-m^{*}(\kappa )}\right] & \text{if}\;\bar{m}\in (m^{*}(\kappa ),1). \end{array}\right.$$

**Proof.**

Fix κ>0. Suppose, for a contradiction, that $c_{\kappa }^{*}$ is not a monotonically decreasing function of $\bar{m}$ on [0,1). Then, we can find $0\leq {\bar{m}}^{\prime }<{\bar{m}}^{\prime \prime }<1$ such that $c_{\kappa }^{*}({\bar{m}}^{\prime })<c_{\kappa }^{*}({\bar{m}}^{\prime \prime })$. Now, choose $c\in (c_{\kappa }^{*}({\bar{m}}^{\prime }),c_{\kappa }^{*}({\bar{m}}^{\prime \prime }))$. Then, there exists a solution of (2.11*a*–*c*) that satisfies the asymptotic conditions (3.3), stays in region $D_{{\bar{m}}^{\prime }}$ and converges to $\left(0,0,{\bar{m}}^{\prime }\right)$ as y→+∞, but there does not exist a solution of (2.11*a*–*c*) that satisfies the asymptotic conditions (3.3), stays in region $D_{{\bar{m}}^{\prime \prime }}$ and converges to $\left(0,0,{\bar{m}}^{\prime \prime }\right)$ as y→+∞. As ${\bar{m}}^{\prime \prime }\in ({\bar{m}}^{\prime },1)$, remark 3.16 gives us a contradiction, hence $c_{\kappa }^{*}$ is a decreasing function of $\bar{m}$ on [0,1).

From lemma 3.14 and conjecture 3.17, we know that the minimal wave speed for all $\bar{m}\in [0,m^{*}(\kappa )]$ is $c_{\kappa }^{*}(\bar{m})=2\sqrt{1-\bar{m}}$. Since $c_{\kappa }^{*}$ is a decreasing function of $\bar{m}$ on [0,1), we must have $c_{\kappa }^{*}(\bar{m})\leq 2\sqrt{1-m^{*}(\kappa )}$ for any $\bar{m}\in (m^{*}(\kappa ),1)$. Finally, by lemma 3.14, we know that $c_{\kappa }^{*}(\bar{m})\geq 2\sqrt{1-\bar{m}}$ for any $\bar{m}\in (m^{*}(\kappa ),1)$. This completes the proof of lemma 3.19.

While we do not have a complete characterization of the minimal wave speed for all κ>0 and $\bar{m}\in (0,1)$, we can now state our second main result. It can be proved similarly to theorem 3.13, so we refer the reader to [5] for its proof.

**Theorem 3.20.**

*Suppose conjecture 3.17 is true. Given* κ>0, *for any* $\bar{M}\in [0,1)$, *there exists a minimal wave speed* $c_{\kappa }^{*}(\bar{M})$ *defined by* (3.25) *such that*:

(i) *For* $0<c<c_{\kappa }^{*}(\bar{M})$, *system* (1.3) *has no weak TWS* (N,M;c) *connecting* (1,0) *and* $\left(0,\bar{M}\right)$.
(ii) *For* $c\geq c_{\kappa }^{*}(\bar{M})$, *system* (1.3) *has a smooth weak TWS* (N,M;c) *connecting* (1,0) *and* $\left(0,\bar{M}\right)$. *Moreover, this solution is unique* (*up to translation*) *and* N,M *are monotonically strictly decreasing and increasing functions of* x−ct=ξ∈R∪{−∞,+∞}, *respectively*.

## 4. Numerical solutions of the PDE model

In this section, we present numerical solutions of the PDE model (1.3), which complement our travelling-wave analysis. We solve (1.3) on the one-dimensional spatial domain X:=[0,L], where L>0, using the method of lines. A detailed description of the numerical methods employed is provided in electronic supplementary material, S2. Similarly to [16], we assume that the tumour has already spread to a position x=σ<L in the tissue and we impose initial conditions that satisfy, for $\bar{M}\in [0,1]$,

4.1 $$\left\{ \begin{array}{ll} N(x,0)=1,\;M(x,0)=0, & \text{if}\;0\leq x<\sigma -\omega ,\\ N(x,0)=\exp\left(1-\frac{1}{1-{\left((x-\sigma +\omega )/\omega \right)}^{2}}\right),\;M(x,0)=\bar{M}(1-N(x,0)), & \text{if}\;\sigma -\omega \leq x<\sigma ,\\ N(x,0)=0,\;M(x,0)=\bar{M}, & \text{if}\;\sigma \leq x\leq L. \end{array}\right.$$

Here, 0<ω<σ represents how sharp the initial boundary between the tumour and healthy tissue is. We complete the mathematical problem by imposing zero-flux boundary conditions for N at x=0 and x=L. We set L=200, σ=2 and ω=1 for our simulations.

**Remark 4.1.**

Initial conditions for N with compact support, such as those given by (4.1), are biologically relevant. We verified that the travelling-wave profile and wave speed are preserved across different initial conditions with compact support for N, i.e. initial conditions of the type of (4.1) (see electronic supplementary material, S2).

### (a) Elucidating the wave speed that emerges in the PDE model

**Figure 3.**
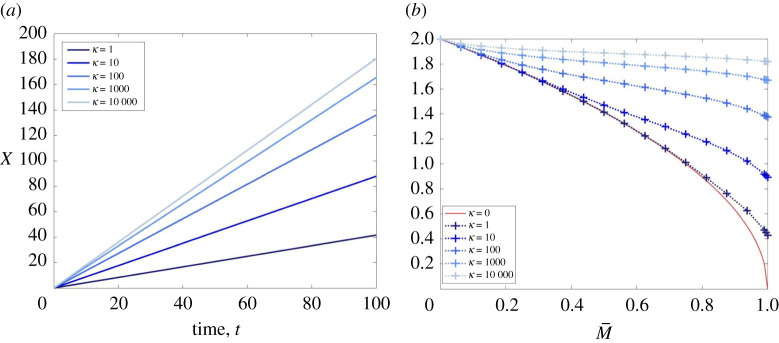
We solve system (1.3) on the one-dimensional spatial domain, x∈X:=[0,200], and impose the initial condition (4.1) for $\bar{M}\in [0,1]$. In (*a*), we plot X(t) such that N(X(t),t)=0.5 for t∈(0,100] in the cases where $\bar{M}=1$ and κ∈{1,10,100,1000,10 000}, and we see that the front travels with a strictly positive, constant propagation speed that increases monotonically with κ. In (*b*), we plot the speed of the travelling front that emerges for $\bar{M}\in \{0.0625j|j\in [[1,15]]\}\cup \{0.99,1\}$ and observe that this speed is monotonically decreasing with $\bar{M}$, given κ>0. (Online version in colour.)

A characteristic feature of the well-studied Fisher–KPP model is that any non-negative initial condition with compact support will evolve towards a travelling front with speed equal to the minimal wave speed, c=2 [37–39]. One may, therefore, question whether this result extends to more complex R–D systems that exhibit travelling waves. For our model, the results from §3e suggest that this does hold for solutions of (1.3) subject to the initial conditions (4.1) for $\bar{M}\in [0,1)$. By contrast, the results from §3d show that there is no strictly positive minimal wave speed for TWS of (1.3) that satisfy the asymptotic conditions (2.2). Yet, the solution of (1.3) subject to the initial conditions (4.1) for $\bar{M}=1$ appears to evolve towards a travelling front with a strictly positive speed, as illustrated in figure 3*a* for different values of κ (see electronic supplementary material, S2 for a travelling-wave profile). In this way, the solutions of the PDE system preferentially select a wave speed in a way that the corresponding ODE system does not.

Given different values of κ>0, we calculated the speed of travelling fronts that emerge for solutions of (1.3) subject to the initial conditions (4.1) with $\bar{M}\in [0,1]$. Our numerical simulations suggest that the wave speed selected by the PDE model is a continuous, decreasing function of $\bar{M}\in [0,1]$, as illustrated in figure 3*b*, which represents this wave speed as a function of $\bar{M}\in [0,1]$ for κ∈{0,1,10,100,1000,10 000}. This is consistent with lemma 3.19 and our observation that the speed of travelling fronts that emerge for solutions of (1.3), subject to the initial conditions (4.1) with $\bar{M}\in [0,1)$, appears to be equal to the minimal wave speed, $c_{\kappa }^{*}(\bar{M})$, defined by (3.25). This result is interesting because the speed selected by the PDE model appears to be left-continuous at $\bar{M}=1$, despite the fact that the minimal wave speed for the existence of TWS is not.

**Remark 4.2.**

Here, we investigated numerically the value of the propagation speed selected by the PDE model. Since the spatial domain, X, must be discretized to solve (1.3) using the method of lines, a natural question is whether the size of the discretization step influences the value of the wave speed. Given TWS of (1.3) that connect (1,0) and $\left(0,\bar{M}\right)$, $\bar{M}\in [0,1]$, we observed that the impact of decreasing the discretization step size becomes significant as $\bar{M}$ approaches 1 (see electronic supplementary material, S2). On the basis of the results illustrated in electronic supplementary material, S2, the discretization step size we used for our numerical simulations ensures that the numerical results obtained are weakly affected by numerical diffusion. In particular, our qualitative descriptions of the wave speed selected by the PDE model are unaffected.

### (b) Comparing trajectories of the PDE and ODE models

**Figure 4.**
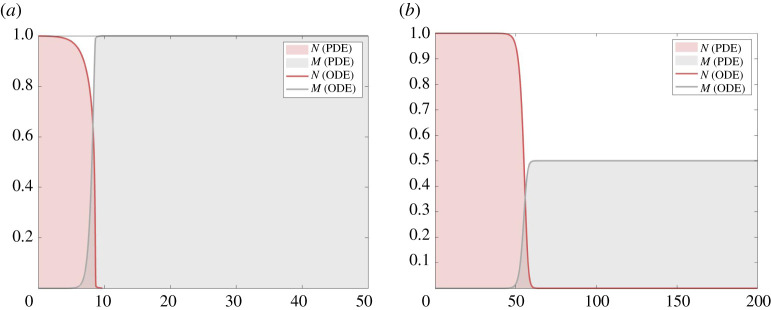
We compare solutions of (1.3), subject to the initial conditions (4.1) with $\bar{M}=1$ (*a*) and $\bar{M}=0.5$ (*b*), with solutions of (2.1*a*,b), subject to the asymptotic conditions (2.2) (*a*) and (2.3) with $\bar{M}=0.5$ (*b*). We use the wave speed estimated from the numerical solution of the PDE model to solve the ODE model and set κ=1. Solutions of the PDE and ODE models agree in both cases. (Online version in colour.)

From theorems 3.13 and 3.20, we know that system (1.3) has TWS connecting (1,0) and $\left(0,\bar{M}\right)$, $\bar{M}\in [0,1]$, for all c>0 if $\bar{M}=1$ and for all $c\geq c_{\kappa }^{*}(\bar{M})$, defined by (3.25), otherwise. Furthermore, we saw that solutions of (1.3) subject to the initial conditions (4.1) for $\bar{M}\in [0,1]$ evolve towards travelling waves and, in particular, that the wave speed is approximately equal to $c_{\kappa }^{*}(\bar{M})$ for $\bar{M}\in [0,1)$. We should therefore be able to find agreement between the wave profiles of the solutions of the PDE system (1.3), subject to the initial conditions (4.1) for $\bar{M}\in [0,1]$, and that of the ODE system (2.1*a*,b), subject to the asymptotic conditions (2.2), if $\bar{M}=1$, and (2.3) otherwise, where we set c to be the wave speed selected by the numerical solution of the PDE system to numerically solve the ODE system. We find good agreement between the wave profiles of the PDE and ODE solutions, and a couple of illustrative examples are shown in figure 4.

## 5. Discussion and perspectives

Understanding the process of tumour invasion is at the forefront of cancer research. The seminal model of acid-mediated tumour invasion developed by Gatenby & Gawlinski [2] generated new biological insights and formed the basis for subsequent mathematical work on this topic. Owing to the model’s complexity, most existing results in the literature on the existence of TWS of the model stem from numerical investigations, which are complemented by partial analytical results. In particular, obtaining a complete understanding of the existence of TWS has proven difficult and this has prompted the derivation of simpler models [15,34]. In this paper, we carried out a travelling-wave analysis for the simplified model (1.3).

We found that system (1.3) can support a continuum of smooth TWS, which are here defined as TWS for which $\bar{\xi }$, introduced in lemma 2.2, satisfies $\bar{\xi }=+\infty$. These TWS represent the invasion of healthy tissue, consisting of ECM, by tumour cells and differ according to the density of ECM far ahead of the wave front. More specifically, we characterized TWS connecting the two spatially homogeneous steady states (1,0) and $\left(0,\bar{M}\right)$, for $\bar{M}\in [0,1]$. Owing to the degeneracy in the first equation of (1.3) for M=1, we distinguished the cases where $\bar{M}=1$ and where $\bar{M}\in [0,1)$.

In the first case, we proved the existence of smooth TWS for any positive wave speed, c>0. This result is particularly interesting as it differs from previous results for degenerate diffusion in a scalar or multi-equation setting, where TWS exist if and only if the wave speed is greater than or equal to a strictly positive minimal wave speed [22,24,25]. It is important to note that this does not imply that a positive wave speed which is preferentially selected does not exist for solutions of (1.3) that connect (1,0) and (0,1). In fact, we saw in §4a that a strictly positive, κ-dependent wave speed appears to be selected by (1.3) subject to the initial conditions (4.1) with $\bar{M}=1$. It would, therefore, be interesting to study the stability of the TWS defined by theorem 3.13. We may gain insight on the minimal wave speed for solutions of (1.3) that connect (1,0) and (0,1) by determining parameter regimes in which solutions are unstable.

In the second case, we proved that smooth TWS exist if and only if the wave speed is greater than or equal to a strictly positive minimal wave speed, $c_{\kappa }^{*}(\bar{M})$, defined by (3.25) for $\bar{M}\in [0,1)$. Given κ>0, this minimal speed appears to be a monotonically decreasing, continuous function of $\bar{M}$. In particular, we conjectured that, given κ>0 and $m^{*}(\kappa ):=1/(\kappa +1)$, we can precisely define $c_{\kappa }^{*}(\bar{M})=2\sqrt{1-\bar{M}}$ for $\bar{M}\in [0,m^{*}(\kappa )]$. Similarly to the generalized Fisher–KPP equation, this minimal wave speed is the smallest c>0 such that the equilibrium $\left(0,0,\bar{m}\right)$, with $\bar{m}=\bar{M}$, of system (2.11*a*–*c*) is a stable node and not a stable spiral. For $\bar{M}\in (m^{*}(\kappa ),1)$, numerical simulations suggested that the wave speed selected by the PDE is strictly greater than $2\sqrt{1-\bar{M}}$, which is consistent with (3.25). The fact that the equilibrium $\left(0,0,\bar{m}\right)$ of system (2.11*a*–*c*) is a stable node is then no longer a sufficient condition to ensure the positivity of the n-component of the TWS in the desingularized variables and thus of the N-component of the TWS in the original variables. This reflects the differences that can be observed in systems of equations compared with scalar equations, which can be attributed to the higher dimensionality of the problem.

Our results regarding the dependence of the minimal wave speed on the model parameters κ and $\bar{M}$ for TWS of (1.3) connecting (1,0) and $\left(0,\bar{M}\right)$, $\bar{M}\in [0,1)$ rely on a conjecture. Our aim is to rigorously prove this result in future work. In addition, we do not have an expression for the minimal wave speed if $\bar{M}\in (m^{*}(\kappa ),1)$. Yet, as κ→+∞, $m^{*}(\kappa )\to 0$, and it is clear that, as κ increases, we can precisely describe the minimal wave speed for a decreasing range of values of $\bar{M}\in [0,1)$. We would therefore like to provide a complete characterization of $c_{\kappa }^{*}(\bar{M})$ for all κ>0 and $\bar{M}\in (m^{*}(\kappa ),1)$. Now, we observed in §4a that the solution of system (1.3) subject to initial conditions (4.1) with $\bar{M}\in [0,1]$ evolves towards a travelling front with a κ- and $\bar{M}$-dependent wave speed. Importantly, given κ>0, it appears that this numerical wave speed is a continuous function of $\bar{M}$ in [0,1], is equal to $c_{\kappa }^{*}(\bar{M})=2\sqrt{1-\bar{M}}$ for all $\bar{M}\in [0,m^{*}(\kappa )]$ and is strictly greater than $2\sqrt{1-\bar{M}}$ for all $\bar{M}\in (m^{*}(\kappa ),1]$. We note that we have included $\bar{M}=1$ in our preceding observations, which highlights our hypothesis that elucidating the minimal wave speed for (1.3) in the case $\bar{M}=1$ could perhaps help us elucidate the minimal wave speed for (1.3) in the case $\bar{M}\in (m^{*}(\kappa ),1)$, or vice versa. It is, therefore, important to also study the stability of the travelling waves defined by theorem 3.20.

Finally, while it is of mathematical interest to obtain a comprehensive description of the minimal wave speed for all TWS of (1.3), it is also of biological interest. Our results indicate that the minimal wave speed is highly dependent on the value of κ, which is the rescaled ECM degradation rate. Since this parameter represents, in a sense, the aggressivity of the tumour cell population towards the ECM, it is significant from an oncological perspective. Hence, our results have the long-term potential of revealing promising targets for therapeutic intervention.

## Data accessibility

Matlab codes to numerically solve the models are available in the electronic supplementary material.
